# Signal Detection and Monitoring Based on Longitudinal Healthcare Data

**DOI:** 10.3390/pharmaceutics4040607

**Published:** 2012-12-13

**Authors:** Marc Suling, Iris Pigeot

**Affiliations:** BIPS-Institute for Epidemiology and Prevention Research, Achterstr. 30, 28359 Bremen, Germany; E-Mail: pigeot@bips.uni-bremen.de

**Keywords:** bayesian signal detection, confounder adjustment, disproportionality analysis, longitudinal data, pharmacovigilance, signal detection, spontaneous reporting, surveillance techniques

## Abstract

Post-marketing detection and surveillance of potential safety hazards are crucial tasks in pharmacovigilance. To uncover such safety risks, a wide set of techniques has been developed for spontaneous reporting data and, more recently, for longitudinal data. This paper gives a broad overview of the signal detection process and introduces some types of data sources typically used. The most commonly applied signal detection algorithms are presented, covering simple frequentistic methods like the proportional reporting rate or the reporting odds ratio, more advanced Bayesian techniques for spontaneous and longitudinal data, e.g., the Bayesian Confidence Propagation Neural Network or the Multi-item Gamma-Poisson Shrinker and methods developed for longitudinal data only, like the IC temporal pattern detection. Additionally, the problem of adjustment for underlying confounding is discussed and the most common strategies to automatically identify false-positive signals are addressed. A drug monitoring technique based on Wald’s sequential probability ratio test is presented. For each method, a real-life application is given, and a wide set of literature for further reading is referenced.

## 1. Introduction

Before releasing a newly developed drug to the market, a thorough assessment of the benefit-risk profile of the drug is made. Despite the often vast efforts to get a clear understanding of a compound’s impact on the body system, not every adverse drug reaction (ADR) may be spotted in pre-market studies. Post-marketing strategies to detect unknown, and to monitor suspected, risks are of high importance and have therefore led to the development of a wide variety of methodological approaches to detect and follow up drug safety signals in recent decades. According to the World Health Organization (WHO), a safety signal is defined as reported information on a possible causal relationship between an adverse event and a drug, the relationship being unknown or incompletely documented previously [1]. A more recent definition was given by the Council for International Organizations of Medical Sciences (CIOMS) [2]: “Information that arises from one or multiple sources (including observations and experiments), which suggests a new potentially causal association, or a new aspect of a known association, between an intervention and an event or set of related events, either adverse or beneficial, that is judged to be of sufficient likelihood to justify verificatory action.” Here, terminology is used that addresses the inclusion of other sources of information besides ADR reporting, reinforces the notion that a possible safety risk is only suggested, and takes into account the needed verification of the potential association between drug exposure and clinical event [3]. Both definitions interpret the term “signal” as a “signal of disproportionate reporting” as proposed by Hauben and Reich [4], not a signal or “alert” as known from a clinical context, where an underlying causality is strongly suspected and has undergone clinical review. This interpretation will also be used throughout this paper.

During the last decades, post-marketing drug surveillance systems in pharmacovigilance relied and still largely rely on spontaneous reporting (SR) data. Maintained by international and national institutions as well as manufacturers, SR databases consist of voluntary reports of serious ADRs after or during drug exposure provided by health care professionals, patients or the pharmaceutical industry, whenever an association between an exposure to a drug and the observed event is suspected [5,6]. Several data-mining techniques have been suggested and used to scan these data collections for signals. SR databases are subject to certain well-documented limitations, such as under-, over- and duplicate reporting, limited information on concomitant medication, or comorbidities and the inability to provide the denominator, *i.e.*, the number of subjects actually consuming the drug of interest [7,8,9].

To overcome some of these limitations, and triggered by several severe safety issues in the last years [10,11,12,13], programs have been initiated to make beneficial use of large data pools besides spontaneous reports. An early pioneer in using electronic health care data for routine safety surveillance is the Vaccine Safety Datalink (VSD) [14] that started to study the adverse side effects of vaccines in 1990. In 2007, the U.S. Food and Drug Administration (FDA) has started to establish the Sentinel Initiative [15,16], which aims at the provision of electronic healthcare data consisting of multiple sources like administrative and insurance claims databases. The target of the Sentinel system is to capture data on more than 100 million individuals for active drug safety surveillance. In the wake of this initiative, the Observational Medical Outcomes Partnership (OMOP) [16,17], a public–private partnership with the Pharmaceutical Research and Manufacturers of America (PhRMA) and the FDA, launched by the Foundation for the National Institutes of Health, strives for improvements in monitoring drugs for safety by researching methods that are feasible and useful to analyze existing healthcare databases. As European counterpart the EU-wide “IMI-PROTECT Project”, a large consortium involving the European Medicines Agency (EMA), academic institutions and the pharmaceutical industry, pursues the goal of strengthening the benefit–risk monitoring of medicines in Europe [18,19]. This endeavor includes the improvement of early and proactive signal detection from spontaneous reports, electronic health records and clinical trials. Moreover, the European Union (EU) has funded the EU-ADR project, where techniques were developed that allow mining for adverse drug reactions in electronic health records (EHR) across European countries [20,21].

A variety of data-mining methods to detect unknown safety signals in SR databases has been developed and established over the last decades, and new methodology that takes advantage of the available longitudinal observational data is nascent. Furthermore, a multitude of drug surveillance techniques has been proposed and tested. The goal of this paper is (1) to give a brief overview of the signal detection process; (2) to describe how different sources of data can be used; (3) to present data-mining methods widely used today, to describe sophisticated methods that were recently developed especially for the use in longitudinal data and to highlight some of the surveillance techniques; (4) to give an outlook on further methods and resources and (5) to discuss some of the real-world applications of the presented methods.

Safety signals may also arise from literature reviews or from findings of pharmacoepidemiological studies, e.g., cohort studies. Such studies are beyond the scope of this paper although they may be set up on pharmacoepidemiological databases.

This paper is organized as follows: Section 2 gives an overview of the workflow in the signal detection process. Section 3 does not only present well-established data sources and data sources that recently became of interest for drug safety research, but also some strategies to prepare the different data sources to make them suitable for signal detection. In Section 4, we discuss the most common signal detection algorithms. We start by introducing simple frequentistic methods designed for SR data and highlight their shortcomings, especially when applied to data with low event counts. These shortcomings can be overcome by more advanced Bayesian techniques especially when frequency counts are low. These techniques, however, ignore possible interactions between several drugs and potential safety hazards, but they can be modified to also cope with this situation as will be shown. Next, the Bayesian measures will be extended to longitudinal data by taking advantage of the information on the duration of drug exposure in the data. Finally, some techniques are presented that do not originate from the SR context and have been developed to take full advantage of longitudinal information in the data. For each of the introduced methods, an application is presented and described briefly. After discussing the problem of confounder adjustment in Section 5, we address the “triage” step, where strategies are applied to automatically separate false-positive signals from those signals that may indicate a safety hazard. Section 7 gives an outlook on techniques on how to monitor potential safety signals over time, before a definite decision can be made whether to discard the signal or to pursue it further. We close with a discussion in Section 8.

## 2. General Workflow

**Figure 1:**
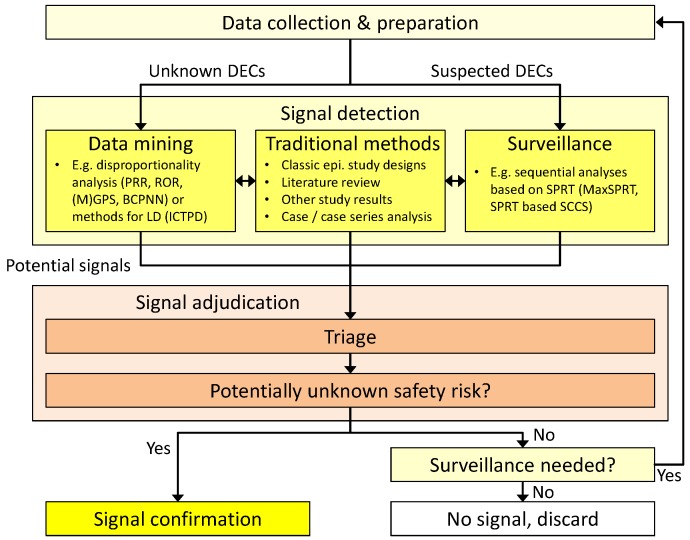
Schematic overview of the signal detection process (based on a figure from [2] in [3]) (DEC = drug-event-combination, PRR = proportional reporting rate, ROR = reporting odds ratio, MGPS = Multi-item Gamma-Poisson Shrinker, BCPNN = Bayesian confidence propagation neural network, LD = longitudinal data, ICTPD = information component temporal pattern discovery, SPRT = sequential probability ratio test, SCCS = self-controlled case series).

The process of signal detection comprises several phases (*cf*. Figure 1). The basis and one of the most crucial parts is the collection and preparation of the data to be analyzed. Different types of data with a variety of available information can be used, ranging from spontaneous reports of ADRs to detailed information from Electronic Medical Records (EMRs). Based on these data, signal detection analyses are conducted. We can coarsely distinguish between two different strategies to detect safety hazards:

(1) Data-mining techniques that strive to uncover so far unknown and unsupected associations. These methods are usually applied to a broad range of combinations of drug exposures and subsequent adverse events, often without limiting the search to pre-defined drug classes or specific medical conditions. They can be regarded as a broad search over the whole spectrum of drug-event combinations (DECs) in the underlying dataset.
(2) If the data-mining search has indicated a possible health risk with a certain DEC, it may be advised to closely monitor this DEC over time to decide whether it should be considered further in confirmatory studies. Surveillance techniques have been developed to consolidate knowledge on these already suspected DECs and are often applied after the first data-mining step.

After the detection of potential signals in the data-mining process, they have to be adjudicated thoroughly to identify all DECs that (a) are already known and well documented; (b) occur very seldom or (c) are highly implausible from a medical perspective and thus can be regarded as artificial false-positive signals. This triage process is crucial in the entire signal detection process as, on the one hand, it can drastically reduce the workload in the following steps, but on the other hand, it can also lead to the dismissal of correctly identified safety signals. Subsequently, each remaining potential signal has to be classified regarding its safety risk. This can either lead to immediate action like confirmatory studies that can result in halt of marketing or even withdrawal of the drug, or given a non-negligible but not critical risk—the decision to closely monitor the DEC via surveillance analysis techniques, or to discard the potential signal as non-hazardous.

## 3. Data Sources

### 3.1. Overview

During the last years, the awareness of the need for extensive drug safety surveillance increased. Programs were set up for a multitude of different data sources and studies were conducted to ascertain whether these data could be of use to monitor unknown effects of drug exposure with signal detection methods.

A wide variety of data sources is considered today [3], such as

- spontaneous reporting databases, like the WHO International Database [22], maintained at the Uppsala Monitoring Centre (UMC) in Uppsala, Sweden; the European Union Drug Regulating Authorities Pharmacovigilance database (EudraVigilance) or a multitude of national databases, e.g., the Adverse Event Reporting System (AERS) as part of MedWatch, the FDA Safety Information and Adverse Event Reporting Program, or the Vaccine Adverse Event Reporting System (VAERS);
- captured data of drug dispensing, e.g., by the New Zealand Intensive Medicines Monitoring Programme [23];
- longitudinal administrative or claims databases from health insurance institutions, like the Medicare database, based on the social insurance program in the U.S., or the German Pharmacoepidemiological Research Database (GePaRD) [24];
- EMRs databases, like the General Practice Research Database (GPRD) in the UK, or data from EHRs, or the Vaccine Safety Datalink (VSD) project [14].

Additionally, types of data that were not available for signal detection before can now be exploited, such as laboratory measurements stored in EMRs. In a recent study by Park *et al.* [25], the authors presented a novel algorithm for detecting signals of ADRs using EMR data with focus on laboratory abnormalities after drug exposure, and to evaluate the potential use of this method as a signal detection tool. Moreover, information sources like medical internet forums or text mining in biomedical literature [26] are becoming of interest. In general, it can be stated that nearly every data source that contains information on health status and drug exposure can be of beneficial use for signal detection purposes.

### 3.2. Spontaneous *vs.* Longitudinal Data

**Figure 2:**
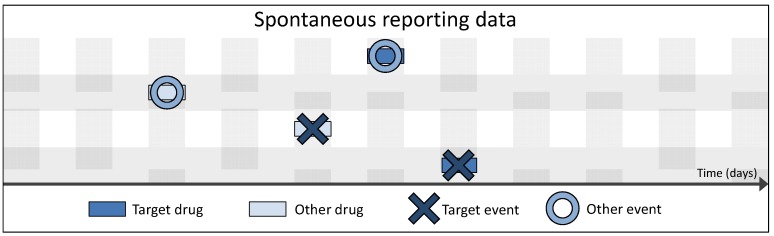
Schematic overview of the amount of information per patient contained in spontaneous reporting data.

SR data are a valuable source of information, as the existing data collections cover very long time periods. Furthermore, national spontaneous reporting systems are installed in numerous countries besides the international data collections, such that a wide range of coverage is reached with these data, both geographically and population-wide. Nonetheless, SR data can only shed light on small sections of the subject’s medical history; the information contained in SR is punctual, that is, usually focused on the day of the ADR only. Hence, SR data are merely a glimpse through a keyhole without the possibility of seeing the full picture (*cf.* Figure 2). Moreover, as already stated, SR data bear a list of well-known limitations. Since the SR system relies on the reporting of adverse drug reactions in the first place, mainly by physicians, but also the consumers, the data from those sources inherently carry an unknown proportion of under-reporting. The reports on ADRs from studies reported through the pharmaceutical industry are considered more complete. SR data, however, also have the potential for over-reporting, *i.e.*, reports on ADRs that are not solely triggered by the expertise and suspicion of a medical expert, but also influenced by other factors, e.g., extensive media coverage of newly suspected adverse reactions after exposure to a certain drug. The fact that multiple actors may report to the system can lead to duplicate reporting. One of the foremost problems from a methodological point of view is the inability to provide incidence rates: SR data only cover subjects with a drug exposure and a subsequent health event; they do not include data on exposed subjects without an event, so the number of subjects actually consuming the drug of interest is not known. This does not allow for risk assessment as in classical pharmacoepidemiological studies [7,8,9]. A further considerable drawback when using SR data lies in the compliance of the reporters with the reporting system regarding the timely feedback on suspected ADRs. A collection of SR data is of dramatically reduced value if ADRs are reported with a significant delay, as pointed out by Ahmad [27]. Especially high-risk DECs need to be detected as soon as possible, which is only possible if data are provided promptly.

Spontaneous reports are usually aggregated in drug-event report tables (*cf.* Table 1), containing the essential information from the “raw” reports. These tables can be processed to be suited for statistical analyses, but the statistical methodology is technically not limited to this data format, as discussed later in this paper.

**Table 1:** Exemplary structure of data derived from spontaneous reports. The reports were processed to drug-event combinations on subject level (0 = no event/exposure reported, 1 = event/exposure reported).

Sex	Age	Drug 1	…	Drug I	Event 1	…	Event J
f	50	0	…	1	1	…	0
m	80	1	…	0	0	…	1
m	68	0	…	1	1	…	1
…	…	…	…	…	…	…	…

**Figure 3:**
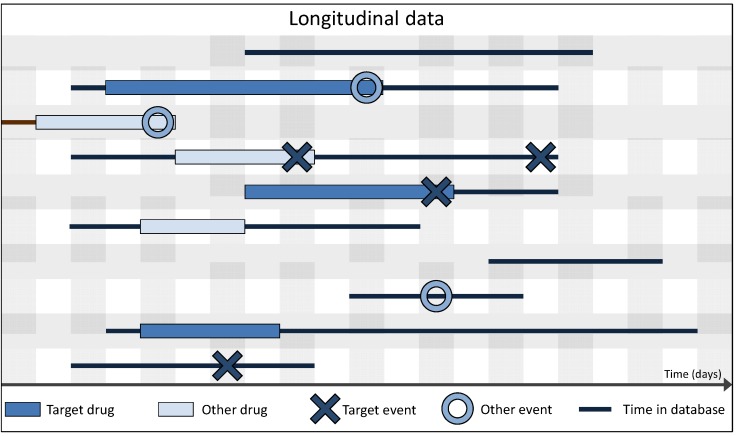
Schematic overview of the amount of information per patient contained in longitudinal data over time, where the information simultaneously contained in spontaneous reporting (SR) data is highlighted.

The use of longitudinal observational data like EHRs or health insurance claims data might widen this narrow view considerably by providing information that is not directly connected with the actual ADR. Although of different origin and different (primary) purpose, these longitudinal data usually bear some key similarities, distinguishing them from SR data: they do not contain duplicates, are typically derived automatically and are, therefore, largely unaffected by under- or over-reporting and they include coherent information on most of the subject’s drug exposure periods (often via outpatient drug prescriptions), clinically relevant events (independently from the exposure status) and—very important—information on exposed subjects without events (*cf*. Figure 3). The assessment of drug exposure and comorbidity status is much more complete as compared to SR data, while this does not automatically imply that 100% of the information is collected. For instance, drug exposures usually are only available for the outpatient setting, information on medications administered during hospitalization periods is often not included. It is also well understood that these routinely collected longitudinal data typically cannot provide information that is not needed for reimbursement, e.g., on lifestyle factors like smoking behavior, alcohol consumption or anthropometric factors like body mass index. This lack of information on lifestyle factors becomes crucial when risk estimates have to be adjusted for potential confounders. A major limitation of most administrative data sources is the lack of validation of the diagnostic coding. As the primary purpose of diagnoses in, for example, claims data is reimbursement, a validation of the coded information against the original source (e.g., a medical chart) is crucial. In the Mini-Sentinel project [28], such techniques for the validation of health outcomes were reviewed on a wide set of outcomes [29]. Additionally, prescription or dispensation data do not contain information of the patients’ compliance to the prescribed drug regimen; the actual drug exposure usually remains unknown.

### 3.3. Data Preparation

The signal detection process starts with data preparation. Until today, many techniques operate on frequencies of DECs, collated in 2 × 2 contingency tables, as given in Table 2. For each DEC *ij* (*i.e.*, drug *i*, ADR *j*) in question, such a table is constructed, e.g., in a database containing information on 15,000 drugs and 15,000 ADRs, 225 million such contingency tables are produced and evaluated during the data-mining process. All subsequent formulae refer to the nomenclature given in Table 2, indices *ij* are omitted where convenient.

**Table 2:** 2 × 2 contingency table containing frequencies of exposure to drug *i* and occurrences of adverse drug reaction (ADR) *j* as basis for signal detection.

Drug *i* × ADR *j*	Event	No event	Total
Exposed	*n*_11_	*n*_10_	*n*_1_
Not exposed	*n*_01_	*n*_00_	*n*_0_.
Total	*n*._1_	*n*._0_	*n*..

The definition of the single cells *n*_11_ to *n*_00_ in the 2 × 2 table highly depends on the level of information contained in the underlying database.

**Figure 4:**
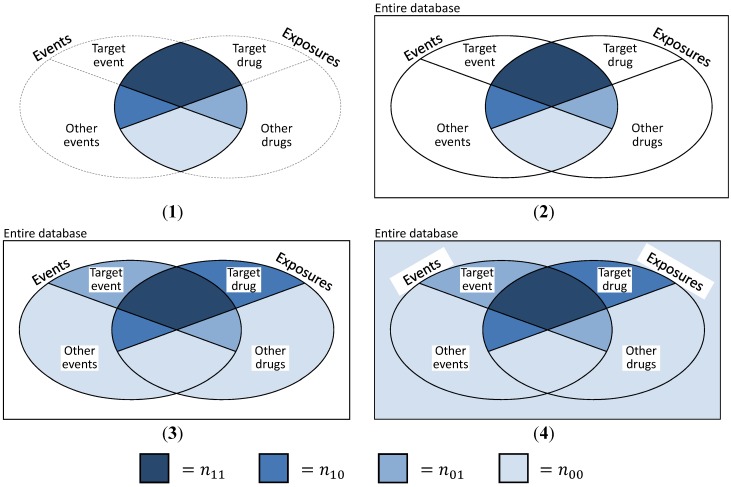
Diagrams to illustrate the different levels of information included when mapping longitudinal data (LD) to 2 × 2 cross-classification tables. (**1**) Structure of genuine spontaneous reporting (SR) data, only events under drug exposure are available; (**2**) “Pseudo”-SR, generated from LD. Only events under exposure are considered, similar to genuine SR data. None of the additional exposure information in LD is used; (**3**) Modified “pseudo”-SR, also including information on events without exposure and exposure periods without event. Some of the additional information contained in LD is used; (**4**) Considering all information available, including exposure- and event-free individuals.

SR data do not contain any information on the unexposed or event-free groups of the general population. Thus, the definitions for “unexposed” and “no event” have to be adapted. “Unexposed” is defined as exposure to any drug besides exposure to drug *i*, “no event” is similarly defined as occurrence of any event apart from event *j*. According to these definitions, *n*_11_ is the number of reports on DEC *ij* and *n*_11_ the number of reports on ADR *j* when exposed to other drugs than drug *i*. Analogously, *n*_10_ and *n*_00_ are defined as the frequencies of reports on other ADRs when exposed to drug *i* or not exposed to drug *i*, respectively (*cf.* Figure 4(1)). In the recent literature [30,31,32,33,34], approaches for the conversion of longitudinal data to 2 × 2 tables were proposed:

(a) The basic and most obvious approach is to create “pseudo”-SRs, trying to simulate the exact data structure presented in Table 1. Then, *n*_11_ is defined as the number of DEC *ij*, *n*_01_ as the number of events *j* while not under exposure *i*, *n*_10_ denotes all exposure periods to drug *i* with a different ADR than event *j* and *n*_00_ is the number of non-*j* events under non-*i* exposure (*cf.* Figure 4(2)). This is a coherent and convenient definition, suffering from the major deficit that information on exposures without events and events without exposures is missing. This approach was discussed and implemented by Schuemie [30] and Zorych *et al.* [35].
(b) Curtis *et al.* [34] proposed a method of converting longitudinal data to SR with the possibility to additionally include information on non-exposures and non-events by introducing temporal segmentation of the data. They considered each month per subject to be a single report, consisting of all events that the subject experienced during this specific month and all drugs that were consumed that month or the month before. Thus, reports similar to the structure shown in Table 1 can be generated, plus reports that might contain information on exposures without events or events without exposures (*cf.* Table 3). Here, *n*_11_ is defined as before and denotes the number of reports on DEC *ij*, *n*_10_ is the sum of all reports on drug *i* without ADR *j*, *n*_01_ is—*viceversa*—the sum of all reports on ADR *j* without exposure to drug *i* and *n*_00_ the number of reports containing neither drug *i* nor ADR *j* (*cf.* Figure 4(3)).
(c) A closely related approach to take advantage of the longitudinal information, but without imitating a “reporting structure” was described by Schuemie [30] and Zorych *et al.* [35]. Here, *n*_11_ is defined as the number of distinct DECs *ij*, *n*_10_ and *n*_01_ stand for the number of all exposure eras to drug *i* without the occurrence of ADR *j* or the number of ADRs *j* not experienced during exposures to drug *i*, respectively. Finally, *n*_00_ is defined as the number of all non-*j* events that occur during non-*i* exposure periods, event-free non-*i* exposure periods and non-*j* ADRs when not exposed to any drug (also *cf.* Figure 4(3)).
(d) The final approach uses even more information than the one presented in c). *n*_11_ is defined as number of individuals experiencing event *j* while exposed to drug *i*, *n*_10_ and *n*_01_ are defined as number of persons with exposure to drug *i* and no occurrence of event *j*, or an experienced event *j* and no exposure to drug *i*, respectively. Finally, *n*_00_ includes all individuals that were neither exposed to any drug, nor experienced any ADR (*cf.* Figure 4(4)). Thus, *n*_11_ + *n*_10_ + *n*_01_ + *n*_00_ equals the number *n* of all subjects contained in the database.

These might not be all approaches currently discussed in the literature, but this overview gives an adequate insight in the basic ideas.

**Table 3:** Exemplary structure of “pseudo” spontaneous reporting data derived from longitudinal databases. Cells with “empty” reports on events or drug exposure, which would not be available in real spontaneous reports are highlighted (0 = no event/exposure reported, 1 = event/exposure reported).

Sex	Age	Drug 1	…	Drug I	Event 1	…	Event J
f	50	0	…	1	1	…	0
m	64	0	…	0	0	…	1
m	80	1	…	0	0	…	1
f	58	1	…	1	0	…	0
m	24	0	…	0	0	…	0
…	…	…	…	…	…	…	…

### 3.4. Definition of Exposure and Event in Longitudinal Data

A crucial step in converting longitudinal data is the definition of what needs to be considered as “event” and “exposure.” 

The estimation of drug exposures depends on the type of available information; often drug prescriptions are considered as a suitable surrogate if exposure information is not directly available. The estimation of the exposure duration is usually constrained by the lack of information on the actual adherence to the prescribed drug regimen. If information on dosing regimen is available, this information can be used to estimate the exposure duration, whereas the number of dispensed units and the defined daily dose (DDD) may be used as substitute [36]; actual drug use can also be estimated from repeated prescriptions when focusing on chronic diseases. A thorough discussion of the definition and calculation of exposed and unexposed person-time is given by Brown *et al.* [37].

An event may be defined as hospitalization due to an emerging illness, an event under drug exposure could then be defined as hospitalization with a preceding drug prescription within a fixed timeframe before the onset of the event. Although this timeframe highly depends on the drug considered, a fixed timeframe usually is chosen for all drugs and events for practical reasons. For instance, Choi *et al.* [31,32] and Kim *et al.* [33] consider 12 weeks before the event to be a suitable timeframe to look for the target drug exposure.

## 4. Analysis Techniques

### 4.1. Overview

Basically, two methodological approaches to detect safety signals can be identified in the recent literature, First, there are the data-mining techniques that operate cross-sectionally on a single data snapshot (non-sequential methods), where the disproportionality analysis methods comprise the most widely applied class of analytical techniques [38]. These methods will be discussed in this section. Second, there are the surveillance methods that sequentially calculate a cumulated risk estimate over a sequence of data snapshots, taken at different points in time (sequential methods), where approaches based on Wald’s Sequential Probability Ratio Test (SPRT) [39,40] are among the applied methods. These methods are typically not applied to detect yet-unknown safety hazards, but to monitor DECs where a health risk is of concern. These surveillance techniques will be reviewed briefly in Section 7 of this paper.

Besides these highly automated methods, traditional approaches like reviews of single cases or case series, literature reviews or results from pharmacoepidemiological studies (e.g., cohort designs or case-control designs) can yield safety signals, but these methods usually need manual input, such as appropriate confounder selection, and are not suited to automatically process large quantities of different drug exposures and ADRs in a reasonable timeframe and are thus not considered here.

As part of the large governmentally funded initiatives mentioned before, development of new methodology in drug safety has taken a big leap forward after the start of the Sentinel Initiative and the OMOP collaborative. Extensive methods libraries with accompanying documentation material were set up, most prominently the documentation regarding the single working areas in the Mini-Sentinel [16,41] pilot program or the OMOP methods library [42]. The latter profited largely from the so called “OMOP cup,” a competition to promote methods development and evaluation of novel algorithms for identifying drug safety issues in observational healthcare data, proclaimed in 2009. 

### 4.2. Disproportionality Analysis Measures for Spontaneous Reporting Data

Disproportionality analysis measures are constructed to identify combinations of drug exposures and ADRs that occur disproportionately often, compared to other drug-event combinations. Originally developed for SR data, disproportionality measures can also be directly applied to longitudinal data. Several different disproportionality measures have been proposed in the literature [35,43,44,45,46,47,48], which can generally be divided into two categories: frequentistic and Bayesian, both relying on the aforementioned 2 × 2 contingency tables. The most popular frequentistic methods are the proportional reporting rate (PRR) [48] and the reporting odds ratio (ROR) [44]; among the Bayesian approaches the Bayesian Confidence Propagation Neural Network (BCPNN) [47] and the Gamma-Poisson Shrinker (GPS) [45] (respectively, its extension, the Multi-item Gamma-Poisson Shrinker, MGPS [46]) are the most prominent and widely used techniques.

As the results of signal detection analyses are usually referred to as “risk” estimates, one might assume that they can be directly compared to risk estimates obtained from case-control or cohort studies in pharmacoepidemiology. This is, however, not the case, as SR data do not contain information on subjects under drug exposure who did not experience a health event, so that “risks” cannot be appropriately estimated. The “risk” estimates obtained from SR data merely serve as a tool to identify the most suspicious signals. These signals then need to be examined further in the signal adjudication step.

#### 4.2.1. PRR and ROR—Simple Measures

The most basic frequentist disproportionality analysis measures that are widely used are the PRR and the ROR. Their estimators are defined similarly to the estimators of the relative risk (RR) and the odds ratio (OR) with:

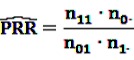
(1)
and

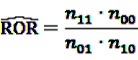
(2)


The limits of the 95% confidence intervals (CI) of PRR and the ROR are usually obtained via an approximation of the normal distribution as

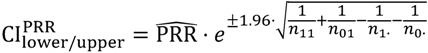
(3)
and

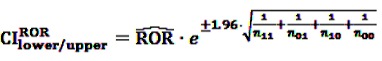
[49]
(4)


The estimates for PRR and ROR are easy to calculate, but the results tend to become unstable when the number of events is small, resulting in large estimates with wide confidence intervals [3,45,50], thus leading to many false-positive signals for very rare events. To uncover these false-positive signals, for instance, the biological plausibility has to be examined and/or confirmatory studies to re-assess the found signals using additional data sources have to be conducted (see also Section 6 of this paper).

As pointed out by van Puijenbroeck *et al.* [44] and Bousquet *et al.* [51], other statistical methods usually applied in cross-classification tables can also be exploited, such as the *X*^2^-test with one degree of freedom (with or without Yates’s correction [52]), where a relevant signal would then require a *X*^2^-value greater than the corresponding 95% quantile 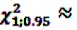 3.85 [50]. Further, rarely applied methods include the crude relative risk (cRR) or Yule’s *Q*-test [53]. The problems that may arise when using such fixed thresholds are discussed in Section 6 below.

#### 4.2.2. BCPNN and GPS—Bayesian Shrinkage

The instability of the above estimators when applied to low drug-event counts led to the development of more advanced Bayesian shrinkage techniques. The two methods mainly used today are the BCPNN, applied at the UMC to analyze the WHO database, and the MGPS, based on the GPS and deployed on the SR data of the FDA.

Both methods are based on the relative reporting ratio (RRR), defined as

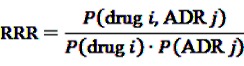
(5)
with *P*(drug *i*) denoting the probability of a target exposure being reported, *P*(ADR *j*) the probability of the target event being reported and *P*(drug *i*, ADR *j*) the joint probability of a report on the target event under exposure to the target drug. In a frequentistic approach, RRR is estimated as

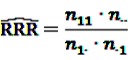
(6)


The 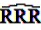 can easily be interpreted: it simply is the ratio of how many ADRs under exposure were actually observed over the number of expected events under the assumption that ADR and drug exposure were independent.

Based on this simple definition of the RRR, the BCPNN estimates the information component (IC), a measure of mutual information between two variables, originating from information theory [54]. It is defined as

IC = log_2_(RRR) (7)

The Bayesian approach underlying the theoretical concept of the BCPNN assumes binomial distributions for both variables and a multinomial distribution for the joint probability. Due to the convenient analytical characteristics of conjugate distributions in Bayesian theory [55], the prior distributions are assumed to be the conjugate priors of binomial and multinomial variables, namely beta distributions. Let 
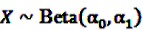
 and 
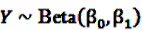
 be random variables to describe the occurrence of drug exposure *i* and ADR *j*, respectively. The *a priori* expectations of *X* and *Y* are obtained as:


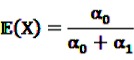
 and 
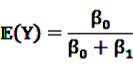


The distribution of the joint occurrence *X* and *Y* is also given as a Beta distribution with

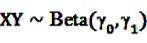

*γ*_0_ = 1, 
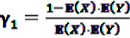
 and an *a priori* expectation of 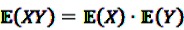, the expected value under the assumption that *X* and *Y* are stochastically independent [56].

After inclusion of the information given in the SR data, a closed form of the *a posteriori* distribution of the IC cannot be given, but the *a posteriori* expectation and the variance of the IC can be derived as

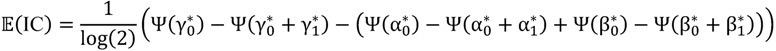
(8)
and

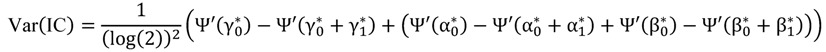
(9)
where 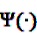 and 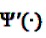 denote the digamma and trigamma function, respectively, and 
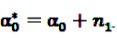
, 
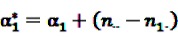
, 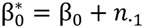, 
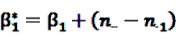
, 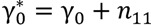 and 
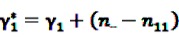
 [56].

An estimate for the credibility interval (CredI) of the IC using Monte-Carlo simulations is given by Norén *et al.* [57].

As an alternative, DuMouchel proposed the so-called Gamma-Poisson Shrinker (GPS) algorithm [45]. Here, the occurrence of the target DEC is considered as rare event such that the observed DEC count *n*_11_ may be assumed as realization of a Poisson-distributed random variable. According to the GPS, the relative reporting rate is defined as

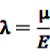
(10)
where *μ* is the mean of the Poisson distribution of *n*_11_ and *E* is the expected event count under the assumption that drug exposure and ADR are independent and is estimated as

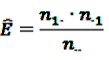
(11)


Following a Bayesian approach, *μ* is not considered as a fixed parameter, but as random, hence *λ* is also considered to be a random variable. The assumption of an underlying Poisson distribution for *n*_11_ leads to a gamma distribution as conjugate prior for *λ*. To add flexibility to the model, a mixture of two gamma distributions with initially unknown mixture parameter *p* is assumed as distribution of *λ*:

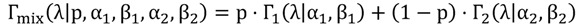
(12)
After calculating the posterior distribution one finds the expectation of log(*λ*) to be

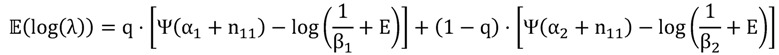
(13)
with

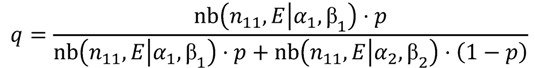

where 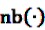 denotes the negative binomial distribution. The resulting risk measure, the so-called “empirical Bayesian geometric mean” (EBGM) is defined as

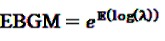
(14)


The EBGM is then estimated by a plug-in approach, where *E* is estimated as shown in Equation 11, and the parameters *α*_1_,*α*_2_,*β*_1_,*β*_2_ and *p* are obtained from the data using an empirical Bayes approach. The fifth percentile of the posterior distribution of *λ* is denoted as “EB05” and interpreted as the lower one-sided 95% confidence limit for the EBGM, the upper one-sided 95% CI is defined analogously as 95th percentile. This Bayesian estimator gives more conservative risk estimates when event counts are small, *i.e.*, the risk estimates are considerably smaller and the CIs less wide, hence the denomination “shrinkage estimate.” While this shrinkage might obfuscate a real signal by reducing it to a non-conspicuous level, it helps to eliminate false-positive signals, which otherwise would have to be adjudicated subsequently.

Most of the aforementioned analysis techniques (PRR, ROR, BCPNN, GPS) are implemented in the “PhViD” package [58] for the statistical software R [59].

#### 4.2.3. Extension of the GPS: the MGPS

The techniques discussed so far assess the risk of 2-way DECs, *i.e.*, one drug and one ADR. Another serious concern is due to potential interactions between several drugs taken simultaneously in relation to the occurrence of an ADR. For the sake of simplicity, let us assume that we are interested in a specific ADR, denoted as *A*, and in two drugs *D*_1_, *D*_2_, where neither exposure to *D*_1_ nor *D*_2_ alone results in an elevated risk for *A*. If the joint exposure to both drugs poses a safety risk, this risk would not be detected in two-way analyses. A famous example is the interaction of cerivastatin and gemfibrozil, leading to an elevated risk of rhabdomyolysis and resulting in the withdrawal of cerivastatin [11,60] from the worldwide market in 2001.

DuMouchel and Pregibon [46] introduced the Multi-item Gamma-Poisson Shrinker (MGPS) as an extension of the GPS algorithm in 2001 to deal with multi-item sets of a size *n* > 2 (e.g., *n* = 3; drug-drug-event interactions). The basic idea is to assess how much of the observed frequency of the joint occurrence of the multi-item-set can be explained by the occurrence of all 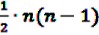 possible two-way interactions in the set of (*n* − 1) drugs under inspection and the event *A* of interest. Given the above set of two drugs (*D*_1_, *D*_2_) and one ADR (*A*), the number 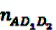 of reports on *A* after simultaneous exposure to *D*_1_ and *D*_2_ is considered to be “interesting” if the number of reports involving the two-way interactions (*i.e.*, *D*_1_ × *D*_2_, *D*_1_ × *A* and *D*_2_ × *A*) does not explain the observed count of the triplet. A log-linear analysis can be conducted to determine if any of the observed frequencies of the two-way combinations depends on the third item. From this analysis one obtains an estimate *e*_AII2F_ of the frequency of reports on the joint occurrence of *D*_1_, *D*_2_ and *A* if all associations were strictly pairwise and independent from the third item. DuMouchel and Pregibon define the EXCESS2 value as number of excess reports on *D*_1_, *D*_2_ and *A* over what might be expected if all associations were only pairwise:

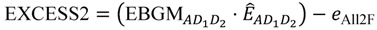



Thus, high EXCESS2 values of an examined triplet might indicate that a safety risk is given under combined exposure to *D*_1_ and *D*_2_. This approach may also be extended to higher interaction levels [46]. A variety of approaches to properly handle multi-item associations can be found for example in von Puijenbroek *et al.* [61,62], Norén *et al.* [57,63], Almenoff *et al.* [43] or Madigan *et al.* [38]. 

### 4.3. Analysis Techniques for Longitudinal Data

#### 4.3.1. Adaptation of the MGPS for Longitudinal Data: the LGPS

For the analysis of longitudinal observational data (e.g., claims data), one option is to convert the data structure to match the structure of SR data, so that the aforementioned techniques can be applied directly. An additional option is to modify the algorithms to better fit the structure of longitudinal data and take full advantage of the available information.

Key information in longitudinal studies includes the number of days a patient was under risk, *i.e.*, the number of days the patient was exposed to the target drug. Let *t*_1_ denote the number of days the patient was under risk, *t*_0_ the number of days the patient was observed without being under risk, and *n*_01_ the number of ADRs *j* when not exposed to drug *i*. Then, according to Schuemie [30] the expected number (*E*) of DECs *ij* can be estimated as:

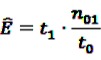
(15)
assuming that the risk is time-invariant. Replacing 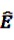 from Equation 11 by Equation 15 to get the plug-in estimator of EBGM (Equation 14) leads to the “longitudinal” GPS (LGPS) algorithm suggested by Schuemie [30]. 

#### 4.3.2. Self-Controlled Case Series

The “self-controlled case series” (SCCS), introduced by Farrington [64] in 1995, can be used to study the temporal association between a time-varying drug exposure and an adverse event using data from cases only. The basic idea of the SCCS is to compare the incidence rate of the event of interest in periods when the individual was exposed to the drug of interest to periods without such exposure. Each case acts as its own control, thereby controlling for both measured and unmeasured confounding variables that do not vary over time, which is the key advantage of the method. Initially developed to study the risk profile of vaccines, it gained wide recognition when used to examine the effect of vaccination for mumps, measles and rubella (MMR) on autism [65]. 

For each individual *k*, risk periods *m*,*m* = 1,2,..., are identified from the data, *i.e.*, windows of time either during or directly after drug exposure. Any other time periods in the observation period for subject *k* are considered to constitute the control periods, indexed by *m* = 0. Moreover, the observation period for individual *k* is split into age groups *l* to incorporate the effect of aging. The occurrence of events is assumed to follow a non-homogeneous, age-dependent Poisson process. 

Let *n_klm_* be the realization of a Poisson-distributed random variable *N_klm_* and denote the number of target events experienced by individual *k* while having spent the time *e_klm_* in age group *l* and risk period *m*. The incidence rate in each such interval, denoted by *λ_klm_*, is assumed to be constant and is given by:

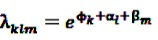
(16)
where 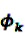 is an effect for individual *k*, *α*_1_ is an effect for age group *l*, and *β_m_* is an effect for risk period *m*. For the baseline period it is assumed that *α*_0_ = *β*_0_ = 0, so that the baseline incidence *λ*_*k*00_ is simply 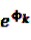.

The number of events *N_klm_* occurring within an interval of length *e_klm_* is assumed to be Poisson-distributed with rate *e_klm_λ_klm_*. The exponentiated quantities 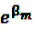 are referred to as relative incidences and are a measure of incidence in risk period *m* relative to the control period *m* = 0.

Conditioning on the total number of events 
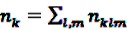
 observed for the individual *k* during the observation period, the log likelihood is multinomial:

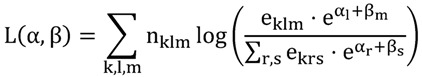
(17)
The method is self-controlled since the individual effects including any time-invariant confounders or random effects, the 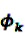, cancel out in Equation 17. Only age (or other time-dependent covariates) need to be modeled. The model 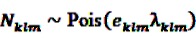 with log link function 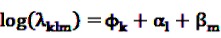 can be fitted using standard statistical analysis software such as STATA^®^ [66] or SAS^®^ [67].

Hocine *et al.* [68] based a technique for near real-time drug surveillance on the SCCS (see Section 7.3). Besides the SCCS, other case-only methods are also used in pharmacovigilance, but not as prominently as the SCCS. A variety of case-only approaches was discussed by Maclure *et al.* [69] with respect to their usefulness for safety monitoring.

#### 4.3.3. IC Temporal Pattern Discovery

Norén *et al.* [70] introduced a technique to identify patterns in the temporal association between the prescription of a drug and the occurrence of a medical event, the “IC temporal pattern discovery” (ICTPD). The method is, very similar to the SCCS, based on the intra-personal comparison of a risk period and a preceding control period. A main difference to the SCCS technique, though, lies in the additional use of information from non-cases, as the ICTPD is mainly focused on the exposure to a certain drug. The methodology is based on a disproportionality approach such as presented in the previous section, where the observed number of ADRs *j* in a certain time period *t* is contrasted to an expected number based on the overall frequency of the ADR relative to other drugs, which is analogous to the approach in Equation 5, but dependent on the time-window *t*. Thus, let further 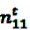 denote the number of prescriptions of drug *i* with a subsequent event *j* in the time-window *t*, 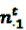 the number of prescriptions to any drug with a subsequent event *j* in time-window *t*, 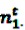 the number of prescription of drug *i* and any subsequent event in time period *t*, and 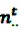 the number of any prescription, followed by any event in time period *t*. Then the IC, defined in Equation 7, for the time window *t* can be estimated as

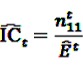
(18)
with

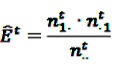
(19)
As described before, the ratio in Equation 18 is sensitive to small event counts, thus, Norén *et al.* [70] proposed a modification of the 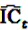 by adding 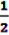 to both the nominator an the denominator, resulting in a general shrinkage towards 0.

Based on this definition of the IC, the authors constructed a measure of temporal association. Let *u* be the time period of primary interest, *i.e.*, the time period after a current drug prescription of drug *i*, and *v* a control time period, against which *u* is to be contrasted. Considering the difference

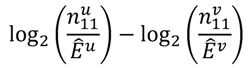
(20)
and rewriting it, the measure IC_Δ_ can be estimated as

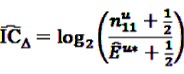
(21)
with

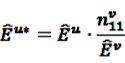
(23)
This estimate 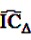, the shrunk difference between the log observed-to-expected ratios in time periods *u* and *v*, can distinguish true temporal associations between an exposure to drug *i* and the occurrence of an ADR *j* from a potential tendency of the joint occurrence of *i* and *j* in the same patients. 

For further information, e.g., on choices of *u* and *v*, the possibility of stratification to adjust for confounding and a sophisticated graphical representation of the IC_Δ_ estimates (the so-called “chronographs”) and their interpretation, we refer to Norén *et al.* [70].

### 4.4. Applications

In the following, sample applications for each analysis technique are presented.

#### 4.4.1. Application of the Disproportionality Measures

Several recent studies used data from the Korean Health Insurance Review and Assessment Service (HIRA), a database including all claims data and prescriptions from approximately 50 million Koreans. In 2010, Choi *et al.* [31] examined the risk profile of rosuvastatin compared to other statins in a population of approximately 1.1 million elderly patients (≥65 years), based on nearly 12 million drug prescriptions by calculating the crude relative risk (cRR) as signal detection method. Target outcomes were several clinical conditions associated with statin use, divided in ADRs for rosuvastain and all other statins. They found 25 signals for rosuvastatin, including 8 that were expected only for this drug, 11 that were expected only for other statins and 6 that were not expected for any of the statins investigated. They considered the cRR measure to be an efficient data-mining tool.

In 2011, Choi *et al.* [32] used the same data to assess the risk of analgesics and anti-inflammatory drugs (AAIDs) in the same cohort of elderly patients. A wider set of signal detection methods was applied: PRR, ROR, BCPNN and the cRR. According to their results, the cRR seems to be the measure with the highest sensitivity and specificity.

Zorych *et al.* [35] used simulated data generated with the Observational Medical Dataset Simulator (OSIM) [71,72] plus anonymized Thomson Reuters MarketScan databases [73]: the lab data (MSLR), the Medicaid Multi-State database (MDCD), the Medicare Supplemental and Coordination of Benefits database (MDCR), and the Commercial Claims and Encounters database (CCAE). They did not focus on specific events or drugs, but ran database-wide analyses across all events and drugs present in the data using the four most common data-mining techniques: PRR, ROR, BCPNN and MGPS. According to the results from the simulated as well as from the real data Bayesian techniques showed better performances than PRR and ROR.

#### 4.4.2. Application of the LGPS

Schuemie [30] also used data generated from the OSIM and real-life data from the Integrated Primary Care Information (IPCI) [74] database from the Netherlands. He focused on detecting the signals that were artificially defined during the simulation process. He applied the longitudinal LGPS algorithm in comparison to other variants of the GPS, and LGPS showed the best performance. Schuemie considered his approach to be highly suitable for signal detection, which is underpinned by the fact that he won the aforementioned OMOP cup in 2010 with the LGPS approach. The LGPS is also successfully applied by the EU-ADR consortium, that examines the combined healthcare data of eight European databases [21,75] for safety signals.

#### 4.4.3. Comparative Studies

Two recent studies by Schuemie *et al.* [75] and Ryan *et al.* [76] compared a wide set of methodological approaches for risk identification in longitudinal data. Schuemie *et al.* assessed the performance of ten different methods, ranging from the simple PRR and ROR to elaborated techniques like the LGPS or SCCS on a dataset comprising information from seven European health care databases, including over 20 million individuals. They examined for each method its ability to detect pre-defined known signals and associations unlikely to represent an ADR (negative controls). Additionally they applied a technique to assess protopathic bias (“LEOPARD”, presented by Schuemie [30], which is briefly discussed in section 6 of this paper) in combination with each of the ten signal detection techniques. They found that the performance of the methods did not differ too much, but that the LGPS and a matched case-control design outperformed the other approaches, whereas the techniques originally designed for SR data fell behind slightly. The filtering for protopathic bias increased the performance for each of the ten methods under inspection.

An analogous approach was made by Ryan *et al.* [76], who compared eight out of 13 analytical methods that were implemented during the OMOP, e.g., disproportionality measures adapted from the SR data analysis, ICTPD, univariate SCCS or a cohort design based on a high-dimensional propensity score (HDPS) stratification [77,78] (*cf.* Section 5 of this paper for details on the HDPS technique). The analyses were based on a combination of various health care databases, covering about 130 million patients. The authors assessed a total of 53 DECs, where nine were true signals and 44 negative controls. For each analysis technique, a variation of method-dependent parameter settings was studied. A set of performance measures was included to rate the performance of the methods, e.g., the area under the receiver operating characteristic (ROC) curve (AUC) [79] or the average precision (AP) [80]. The authors found the cohort design based on HDPS adjustments to be the most predictive, followed by the ICTPD and the univariate SCCS. Similar to the results of Schuemie *et al.*, the disproportionality measures originating from the SR data analysis were among the least predictive models. All assessed methods that were developed in the OMOP are freely accessible in the OMOP methods library [42].

## 5. Confounder Adjustment

A largely unsolved problem is the adjustment for confounding, as pointed out by Lu [81] or Hauben and Bate [9,50]. It is important to ensure that confounding does not produce spurious safety signal warnings of non-existing hazards. With longitudinal data, information on some relevant confounding factors is at hand (e.g., concomitant medication or comorbidities), although other potential confounders are usually still lacking, such as smoking behavior or alcohol consumption. Adjustment for underlying confounding is not *per se* part of signal detection techniques, especially not of the simpler ones (PRR, ROR), but can partially be achieved by stratification for certain variables, like sex and/or age (groups). The disproportionality measures can then be calculated per stratum, although stratification usually comes at the cost of more sparse data tables, thus jeopardizing the stability of the statistical analyses if event counts are low. 

To adjust study results for confounders, a lot of sophisticated approaches exist for pharmacoepidemiological study designs, which can, however, not easily be exploited for signal detection methods in the pharmacovigilance context. As a major problem, the selection of the appropriate confounders that have an impact on the outcome is usually a manual process, involving expertise from various scientific disciplines. For high-throughput signal detection methods this is not feasible, and automated solutions would be needed. This problem was addressed by Schneeweiss *et al.* with the introduction and application of high-dimensional propensity scores (HDPS) [77,78], a technique based on the propensity score (PS) introduced by Rosenbaum and Rubin [82]. The HDPS algorithm automates the selection of potential confounders to be considered in a multi-step approach. Briefly, the HDPS algorithm

(1) requires the identification of the different data dimensions (e.g., hospitalization data, outpatient care data, outpatient drug dispensation data) in the database;
(2) identifies a pre-specified number of the top most prevalent codes, e.g., ICD or ATC codes (ICD = international statistical classification of diseases and related health problems, ATC = anatomical therapeutic chemical classification system) in each data dimension as candidate covariates;
(3) ranks candidate covariates based on their recurrence (the frequency that the codes are recorded for each individual during the baseline period);
(4) ranks covariates across all data dimensions by their potential for control of confounding based on the bivariate associations of each covariate with the treatment and with the outcome;
(5) selects a pre-specified number of covariates from Step 4 (e.g., 500) for PS modeling, and
(6) estimates the PS based on multivariable logistic regression using the selected covariates plus any pre-specified covariates.

This technique theoretically allows for a fully automated selection of confounders for each signal detection analysis. Rassen and Schneeweiss [78] applied the HDPS to control for confounding in sequential database cohort studies. They concluded that HDPS offers substantial advantages over non-automated alternatives in active product safety monitoring systems. In the comparative study by Ryan *et al.* [76], the HDPS stratified cohort design turned out to lead to the best predictive model. Garbe *et al.* [83] compared the performance of the HDPS *vs.* conventional PS with manual confounder selection, using data from the German Pharmacoepidemiological Research Database (GePaRD). Here, the comparison of HDPS and conventional PS matching resulted in improved point estimates for the HDPS when studying an intended treatment effect of coxibs *versus* traditional non-steroidal anti-inflammatory drugs, benchmarked against results from randomized controlled trials.

A common approach to adjust for confounders in pharmacoepidemiological studies is the use of a multiple logistic regression model [84]. In the pharmacovigilance setting with often >10,000 covariates present, confounding theoretically can be introduced by any of these covariates. Regression against all of these possible confounders bears (a) theoretical problems and (b) until recently also technical problems in terms of necessary computing time. Although methodology for automated selection of confounders is developing (e.g., HDPS), advanced logistic regression models are also considered. Genkin *et al.* [85] proposed a Bayesian logistic regression (BLR), where millions of confounders can be included in the analysis. The BLR was applied to the WHO SR database at the UMC by Caster *et al.* [86], and they concluded that the BLR does offer practical advantages, as it can eliminate false-positives and false-negatives due to other covariates, and it identifies some established drug safety issues earlier than a measure based on contingency tables.

A different approach to adjust for unmeasured and measured time-invariant confounding is taken in the case-only methods, such as SCCS (*cf.* Section 4 of this paper, see also especially Hocine *et al.* [68] and Maclure *et al.* [69]). Here, as already mentioned, each case is its own control, thereby controlling for both measured and unmeasured confounding variables that do not vary over time, such as sex, genetic predispositions or general state of health.

## 6. Triage—Adjudication of Potential Signals

When the potential signals have been identified by one of the described analytical methods, the impact and the importance of each identified potential signal have to be evaluated. The aim of this adjudication process is the identification of those signals that are likely to indicate a yet-unidentified safety hazard, and the elimination of false-positives from the results. The sheer number of DECs usually under examination, especially when data-mining methods are applied, makes a structured adjudication of the results a necessity.

First, the plethora of potential signals needs to be reduced by application of a set of basic rules. In EudraVigilance, only signals that are based on three cases or more, show a risk estimate of 2 or higher and have a lower confidence limit greater than 1 are considered relevant. A different approach may be based on a *X*^2^-test with one degree of freedom, where a relevant signal would then require a *X*^2^-value greater than 3.85 [50]. Such simple rules can drastically reduce the amount of potential signals to be processed. A different approach to reduce the number of potential signals is not to focus on the risk estimates from the signal detection, but on a fixed number *R* of potential signals that will remain. The list of analyzed DECs is ordered by the size of the respective risk estimates and cut after the top *R* entries. More sophisticated approaches were suggested by Ahmed *et al.* [87,88] where “rules of thumbs,” e.g., any reliance on fixed thresholds and the estimated 95% confidence interval were avoided. Here, the decision criterion relies on the estimation of the false discovery rate (FDR) [89]. Bayesian and non-Bayesian FDR-based methods were proposed that address the arbitrariness of thresholds and allow for a built-in estimate of the FDR. Simulation studies indicated that these methods can be suitable alternatives to the currently used methods; see [88] for more details.

After having reduced the number of potential signals by mere technical restrictions, the remainders need to be assessed on a qualitative level. A common step is to exclude—automatically if possible—all known and well-documented risks and to focus on the unknown or unexpected identified signals. The exact layout of this part of the triage highly depends on a number of factors, including the underlying data structure, the signal detection method used and personnel resources, as in-depth medical and pharmacological knowledge is necessary. No globally standardized modus operandi can be defined, but many institutions assessing drug safety data utilize a defined set of triage criteria. For instance, the WHO criteria applied at UMC have been well documented by Ståhl *et al.* [90], Lindquist [91] and Hauben and Bate [50] and include several different aspects like the public health impact of the outcome, novelty of the drug in the market or comparison of the results with prior analyses. 

Besides these qualitative assessments, other sources of false-positive signals can be identified, for instance cases of protopathic bias [92], when a drug is administered to treat the condition in question, thus leading to reverse causality in the signal detection process. Schuemie [30] proposed a framework to identify signals caused by such a bias, the Longitudinal Evaluation of Observational Profiles of Adverse Events Related to Drugs (LEOPARD). It is based on the comparison of prescription rates of the drug under consideration in time windows before and after the event, and a potential signal is considered to be protopathic if the number of prescriptions after the event is higher relative to the prescriptions before the event.

Once the triage is completed, the safety risk of every remaining signal needs to be rated to decide whether (a) impact analyses and subsequent confirmatory analyses need to be induced; (b) the signal should be monitored to sharpen the risk profile or (c) the signal can be discarded because of low potential risk. Schneeweiss [93] proposed a systematic approach for these confirmatory steps.

## 7. Near Real-Time Surveillance Techniques

If a certain DEC is suspected to bear a safety hazard after the data-mining process, but no certain assertion for an elevated risk can be made yet, it might be advisable to monitor the respective DEC closely. It would be most preferable to assess the risk profile of the active agent of concern in real-time, *i.e.*, whenever new information is available, or in near real-time, *i.e.*, at regular short intervals. For this purpose, classical sequential tests might be applied as e.g., Wald’s Sequential Probability Ratio Test (SPRT). Wald’s approach is based on the simple null hypothesis *H*_0_:RR = 1 *vs.* the simple alternative *H*_1_:RR = r with a known fixed r > 1. Let *X_t_* be a Poisson-distributed random variable representing the number of observed adverse events at time *t* with means *λ_t_* and r*λ_t_* under the null and the alternative hypothesis, respectively, *x_t_* the realization of *X_t_*. Wald’s approach is based on the repeated assessment of the likelihood ratio test statistic

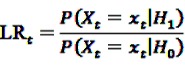
(24)
which results under the above assumptions in

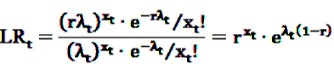
(25)
This can equivalently be written as a log likelihood ratio test statistic

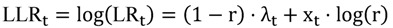
(26)
LLR_*t*_ is calculated at each point in time *t* > 0 as additional data are included. To decide whether the null hypothesis or the alternative is assumed to be true, the obtained value of the test statistic is compared to a lower and an upper critical value. For a given significance level *α* and a power of 1 − *β*, the null hypothesis is accepted if 
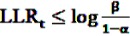
, the alternative is accepted if 
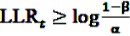
. If LLR_*t*_ lies between these values, no decision can be made and additional data are needed.

This approach is of limited practical value for the assessment of an unknown drug risk for two main reasons. First, the simple alternative *H*_1_:RR = r requires the magnitude r of the elevated risk to be known, which is usually not the case as one is merely interested in an unknown elevated risk RR > 1. Second, the baseline frequency *λ_t_* also needs to be known as it is part of the test statistic. This is not always the case.

### 7.1. Extensions

To overcome the first of these two restrictions, Kulldorff *et al.* [94] introduced the MaxSPRT, extending Wald’s original approach by allowing for a composite alternative hypothesis 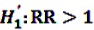. This leads to a modified test statistic, where the denominator is still given by the simple likelihood of the null hypothesis, but the numerator now is given as the maximum likelihood under the composite alternative hypothesis. The likelihood ratio based test statistic is then

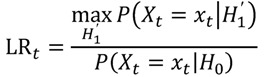
(27)
resulting in

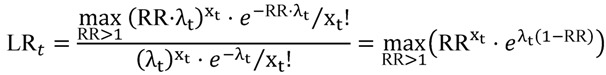
(28)
Replacing RR in Equation 28 by its maximum likelihood (ML) estimator 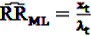 gives

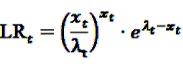
(29)
or

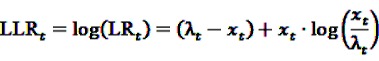
(30)
respectively. Again, the value obtained from the test statistic is compared to lower and upper critical values that have to be derived numerically. Exact critical values for a range of given α and β are provided by Kulldorff *et al.* [94].

To handle the second of the above restrictions, *i.e.*, the often unknown baseline frequency *λ_t_*, one needs access to historical data that were collected before the start of the current surveillance in a cohort not exposed to the drug of interest. From these data, *λ_t_* can be estimated and this estimate can be used in the test statistic via a plug-in approach. While this seems to be reasonable, it inherently creates a new problem: 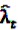 now used in the test statistic is just an estimate, not the real baseline frequency, and as such it has a variability of its own. If we neglect this variability by simply treating 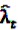 as if it was the real value, we could spoil the results by assigning 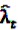 a level of certainty it does not possess.

Li *et al.* [95] thus introduced the so-called Conditional MaxSPRT (CMaxSPRT). *λ_h_* and *λ_s_* denote the event frequencies in the historical data and the surveillance data, respectively. The question that needs to be answered is whether *λ_h_* and *λ_h_* are equal or not, leading to the hypotheses H_0_:λ_s_ = λ_h_ = λ_0_ *vs.* *H*_1_:*λ_s_* > *λ_h_*. The joint likelihood of the historical data and the surveillance data after the *k*^th^ event is given as:

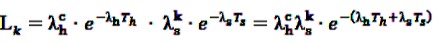
(31)
where *c* denotes the number of events in the historical data and *T_h_* and *T_s_* the person-time in the historical and surveillance data, respectively. The test statistic LLR_*k*_ can now be derived as the log of the ratio of the maximum of the likelihoods under the null and the alternative hypothesis, *i.e.,*

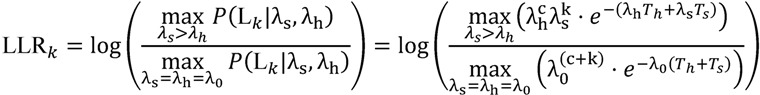
(32)
Replacing the unknown parameters in Equation 32 by their ML estimators 
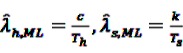
, and 
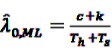
 gives

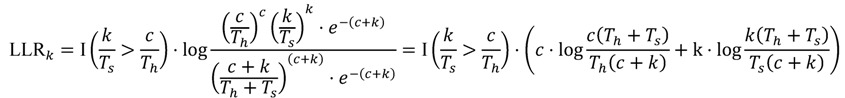
(33)
with indicator function I(·). By conditioning on the numbers of events *c* and *k*, the only remaining random parts in LLR_*k*_ are the person-times *T_h_* and *T_s_*. The critical values can be obtained by Monte-Carlo simulations. Furthermore, this procedure can be extended to also allow for the inclusion of confounding variables and to adjust for the extra person-time after the last observed event in the historical data [95].

### 7.2. Application of the SPRT and MaxSPRT

An application of MaxSPRT was presented by Brown *et al.* [96] who conducted a proof of principle evaluation. They assessed well-documented risks of several drugs (the NSAIDs celecoxib, rofecoxib, valdecoxib, the ACE-inhibitor lisinopril and the statin cerivastatin) plus comparator drugs and two negative controls (cetirizine, clemastine) using data from multiple health plans involved in the Research Network’s Center for Education and Research on Therapeutics (CERT) [97] of the US Health Maintenance Organization (HMO). Analyzing data from approximately eight million insurance members covering a six-year study period, the drug-event associations known from literature were found for four of five drugs, whereas the negative controls triggered no signal. Brown *et al.* discussed several methodological problems using healthcare data for signal detection, they endorsed the use of the MaxSPRT technique in the monitoring of drug safety. Li [98], who also used the CERT data, assessed the risk of acute myocardial infarction (AMI) after intake of rofecoxib compared to diclofenac and naproxen using the CSSP method. The obtained results were consistent with those reported by Brown *et al.* [96].

Davis *et al.* [99] applied a SPRT analysis to the Vaccine Safety Datalink (VSD) data to assess the risk of intussusception after rotavirus vaccination and risks for fever, seizures, and other neurologic adverse events after the change from whole cell diphtheria-tetanus pertussis (DTPw) to a cellular DTP vaccine (DTPa). They detected an increase in intussusception after 2589 vaccine doses of rotavirus vaccine, which was an expected signal, and decreases in fever rates, febrile seizures and other neurologic events with DTPa, where such an association has been suggested by prelicensure trials. The authors concluded that active and prospective surveillance analysis of VSD data provides a valuable, population-based early warning system. 

Near real-time safety surveillance and sequential analysis were pioneered for and have been mostly used for vaccine safety surveillance. Yih *et al.* [100] reviewed a large number of post-market vaccine safety surveillance studies using the Poisson-based maxSPRT described in Section 7.1 and a binomial-based maxSPRT (not described in this paper, see [94] for details).

### 7.3. Further Approaches

Further approaches can be found in the literature as for instance if sequential tests should be applied on a regular basis, say weekly or monthly, where group sequential testing methods might be more appropriate as proposed by Li [98] and Li *et al.* [101].

Hocine *et al.* [68] presented a sequential case-series analysis, combining the idea of the SPRT with the SCCS design. They applied the SPRT, implemented as a group sequential test, to the log-likelihood of the self-controlled case series model. 

Furthermore, Jin *et al.* introduced algorithms that basically strive to rate the “unexpectedness” of an event [102,103]. Alternative techniques for visual inspection have been suggested in [104,105,106,107]. 

## 8. Discussion

In this paper, we gave an overview of the process of signal detection, including available data sources and the preparation of the data, various analysis techniques for SR data, how some of them can be extended to make beneficial use of additional information available in the data and techniques specifically developed for longitudinal data. We briefly addressed the adjustment for underlying confounding as well as the decision making during the triage before presenting methods for drug surveillance.

Looking back on the past decade, Hauben and Bate [9], Hauben and Norén [108] and Lu [81] stated unanimously that disproportionality analyses for signal detection purposes are a worthwhile addition to pharmacovigilance and may be of great value when applied with care, *i.e.*, knowing that the techniques and the underlying data have their limitations. While the usage of longitudinal data for “classical” pharmacoepidemiological studies is routine today [38], the usage of such data for pharmacovigilance is still an emerging field. Impressive amounts of data are potentially available for analysis [21,35,76], and the secondary use of healthcare data for signal detection research is endorsed by many [3,35,109]. It seems plausible to extend the utilization of disproportionality measures from SR to longitudinal data as a first step. Large-scale databases offer vast amounts of information, which can allow for more refined analyses, addressing some of the shortcomings of SR data [35]. To apply methods known from SR data, the longitudinal data need to be transformed to be digestible for the algorithms. As a second step, methods known from SR data can be modified to take advantage of the longitudinal data structure directly. Enhancements especially of the Bayesian techniques have been shown to yield results of higher relevance than the methods for SR data, as indicated by the findings of Schuemie [30] and the combination of the LGPS and LEOPARD algorithms. However, following the results of Schuemie *et al.* [75] and Ryan *et al.* [76], the techniques that originate from the SR data realm often fall short compared to the newly developed techniques for longitudinal data, e.g., those developed or refined in the wake of the OMOP, like ICTPD by Norèn *et al.* [70] or the application of SCCS or HDPS in pharmacovigilance [68,77,78]. 

Madigan and Ryan [109] proposed a set of simple questions, comprising central problems: what methods should be used to yield the best results, how can disparate data collections be combined beneficially, when does a signal need to be considered as a risk, what to do with newly identified risks and how often do the detection methods fail to deliver the correct result? For SR data, some questions seem to be answered: regarding the choice of methods, the “big four” (namely PRR, ROR, BCPNN, MGPS) seem to be the dominant data-mining methods, and according to Madigan *et al.* [40], Bayesian methods have shown clear advantages when applied to SR data and are the *de facto* standard for SR data analysis worldwide today. For drug surveillance, MaxSPRT has gained a lot of attention, as well as case-only methodology [69]. For signal detection on longitudinal data, it is yet unclear which of the existing techniques yields the best result, but the findings of Schuemie *et al.* [75] and Ryan *et al.* [76] suggest that the techniques used in the OMOP, like the combination of LGPS and LEOPARD or the HDPS methodology are likely to provide results of high relevance. However, the methodology for longitudinal data is still nascent, and according to Madigan and Ryan [109] only extensive empirical experimentation can bring answers to the question of the optimal analysis strategy.

Although one might state that the applications presented above showed reasonable results, a few limitations regarding automated analyses should be kept in mind. Generally, Ray [110] claimed that a high level of awareness for the inherent complexity of the studies and the data is needed. A major issue arises from the fact that no true gold standard exists to assess the quality of the results obtained from data-mining analyses [36,80]. One possible way out would be to compare results from a data-mining-study with those from a pharmacoepidemiological study regarding some well-known safety hazards to assess the performance of the applied data-mining technique, although this is, of course, no proper validation. To validate a specific result, the same study could be run on a second database to confirm the first finding. If, however, several large databases are pooled to detect even rare signals, such a finding could possibly not be replicated since equally large databases allowing such a study might not be available [111].

The challenging research field of signal detection comprises many more facets and not all could be discussed in this paper such as the merging of several databases which requires the development of a common data model [112], the mapping of terminology from different coding schemes (diagnostic codes, drug coding, *etc.*) which needs to be adhered to [21,113], the “innocent bystander” effect [9,45], and last, but not least, data protection issues or other legal questions that may arise when different databases containing sensitive patient information are combined [16].
